# An Unexpected Case of Clear Cell Adenocarcinoma of the Cervix in a Non-Diethylstilbestrol-Exposed Postmenopausal Woman

**DOI:** 10.7759/cureus.76083

**Published:** 2024-12-20

**Authors:** Denise Baloi, William J Smith, Robin Samyn

**Affiliations:** 1 Department of Family Medicine, Michigan State University College of Human Medicine, East Lansing, USA; 2 Department of Pathology, Detroit Medical Center, Wayne State University School of Medicine, Detroit, USA; 3 Department of Family Medicine, Corewell Health Beaumont Grosse Pointe Hospital, Roseville, USA

**Keywords:** abnormal vaginal bleeding, cervical cancer screening, clear cell adenocarcinoma of the cervix, diethylstilbestrol, postmenopausal bleeding

## Abstract

Clear cell adenocarcinoma of the cervix (CCAC) is a rare subtype of cervical adenocarcinoma. It has been linked to intrauterine exposure to diethylstilbestrol (DES) but can happen in non-DES-exposed patients, albeit less commonly. Presentation is largely vaginal bleeding, emphasizing the importance of considering CCAC in the differential of abnormal vaginal bleeding despite the tumor’s rarity. We present the case of a non-DES-exposed 68-year-old woman who presented with postmenopausal bleeding (PMB). The patient underwent total abdominal hysterectomy after misdiagnosis as clear cell endometrial adenocarcinoma, later diagnosed as CCAC. We encourage consideration of clear cell adenocarcinoma of the cervix in the differential diagnosis of PMB even in patients without DES exposure and for physicians to advocate for patients to keep up to date with cervical cancer screenings.

## Introduction

Cervical cancer is the fourth most common cancer in females, accumulating a significant global death toll annually of around 350,000 [1]. Clear cell adenocarcinoma of the cervix (CCAC) is a rare histological variant of lower genital tract cancer that only accounts for 4-9% of cervical adenocarcinomas, with around 75% of invasive cervical tumors consisting of squamous cell carcinomas [2,3]. According to Galic et al., adenocarcinoma histology unfavorably affects survival rates in both early and late stages [4]. CCAC has historically been linked to intrauterine exposure to the miscarriage medication diethylstilbestrol (DES), which was no longer administered after 1971 [2]. Still, around 60% of current clear cell adenocarcinoma cases are linked to its exposure, with DES daughters (females whose mothers took DES while pregnant) having a 40 times higher risk of being diagnosed with clear cell cancer than those not exposed [2,5]. Although CCAC development with DES exposure is rare, with one in 1,000 DES daughters being diagnosed, it is still possible for non-DES-exposed females to develop CCAC as well [2]. Presentation is typically vaginal bleeding but can vary, leading to possible misdiagnosis or delayed diagnosis due to the rarity of this tumor [3]. Therefore, it is important to evaluate for CCAC upon presentation of vaginal bleeding in both DES- and non-DES-exposed patients and to encourage timely pap smears for early prevention due to its variable presentation. 

To highlight the rarity of this tumor, we present a case of a 68-year-old woman diagnosed with clear cell adenocarcinoma of the cervix who had no history of intrauterine DES exposure. 

## Case presentation

A 68-year-old female with no significant past medical history presented with postmenopausal bleeding. The patient has an extensive family history of cancer (lung cancer in the maternal grandmother and paternal grandfather, uterine cancer in the mother) and a moderate smoking history of six-pack years of tobacco with limited alcohol use. The patient denied a history of DES exposure in the mother and maternal grandmother, along with risk factors such as endometriosis, previous human papillomavirus (HPV) infection, multiple sex partners, or other sexually transmitted infections. The patient’s last pap smear was three years prior and was due; however, her vaginal bleeding was so light that she almost canceled her appointment. Yet, her primary care physician motivated her to undergo a pap smear, which revealed atypical glandular endothelial cells suspicious for malignancy and was HPV negative by polymerase chain reaction (PCR). One week later, pelvic and transvaginal ultrasound displayed a normal-sized postmenopausal uterus with poor visualization of the endometrium, for which a repeat scan was recommended but not performed. An endometrial biopsy 11 days later showed clear cell endometrial adenocarcinoma and a International Federation of Gynecology and Obstetrics (FIGO) grade of IB1. CT chest/abdomen/pelvis were unremarkable. The following week, the patient underwent a robotic-assisted total abdominal hysterectomy, bilateral salpingo-oophorectomy, bilateral pelvic sentinel lymph node dissection, para-aortic lymph node dissection, and omental biopsy. 

**Figure 1:**
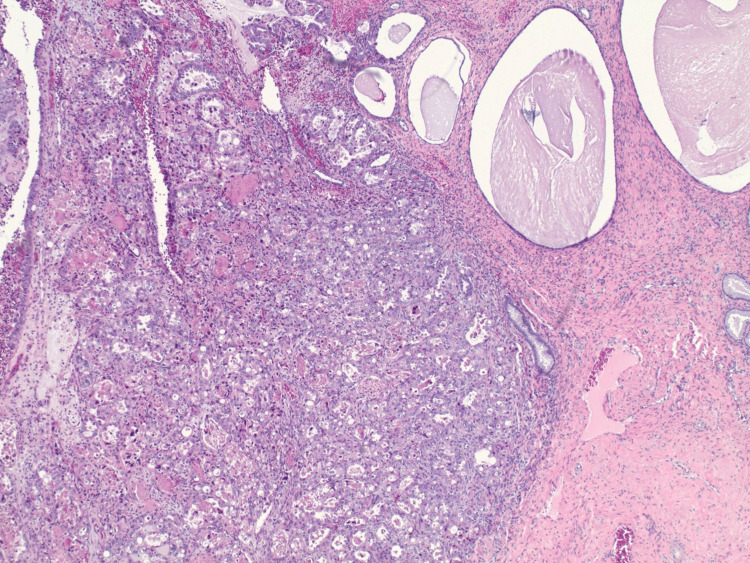
Hematoxylin and eosin (H&E)-stained section, 40x magnification H&E-stained section shows tubulocystic to solid tumor architecture (left) and benign nabothian cysts (right). Tubules are lined by a single layer of cells with eosinophilic to clear cytoplasm with hobnailing, with intraluminal eosinophilic material also present.

**Figure 2:**
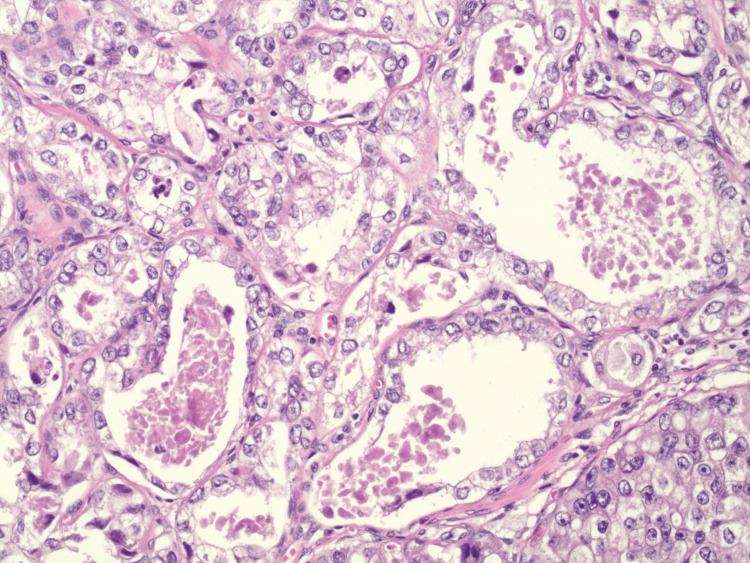
H&E-stained section, 200x magnification This magnification further highlights the nuclear atypia, pale eosinophilic to clear cytoplasm, hobnailing, intraluminal eosinophilic material, and conspicuous nucleoli.

The tumor, arising from the endocervix, measured 1.5 x 1.5 x 0.7 cm and was grade 2. Cervical stromal invasion was 6 mm with a 15 mm horizontal extent, without clear myometrial invasion. No lymphovascular invasion (LVI) was identified. All margins were negative by at least 4 mm, and four sentinel nodes were negative for metastatic carcinoma. The uterus, fallopian tubes, and ovaries were free of tumors, and the omental biopsy was negative. Tumor cells showed immunoreactivity for IMP3 and Napsin A and were not immunoreactive for WT1 or p40, consistent with clear cell carcinoma of the cervix. Pathology showed invasive clear cell carcinoma that was confined to the cervix, not the endometrium (Figures 1, 2). Given that the patient did not have a radical hysterectomy, adjuvant pelvic radiation therapy was recommended to reduce the risk of locoregional recurrence. The patient underwent 27 radiotherapy treatments; follow-up pap smears were unremarkable.

## Discussion

Although the use of synthetic estrogen DES was stopped in 1971, intrauterine exposure to DES still remains a significant risk factor for clear cell adenocarcinoma, which makes timely cervical cancer screenings encouraged for DES daughters [6]. However, it is still possible for patients without exposure and no other risk factors to develop CCAC [3], as was seen in this case report. According to a study completed by Herbst, the median incidence age for CCAC in DES-independent patients was around 51 years (compared to 19 for DES-associated), suggesting it is more frequent in postmenopausal women [7]. This emphasizes the importance of considering CCAC as a differential diagnosis for abnormal vaginal bleeding in postmenopausal women even with no DES exposure history. Though not statistically significant, a study by Reich et al. noted that patients diagnosed with the more common squamous cell carcinoma and non-clear cell carcinoma had a greater five-year survival rate of 80% and 77%, respectively, compared to non-DES-exposed patients diagnosed with CCAC (67%) [8]. This underscores the need for providers to encourage patients to keep up to date with cervical cancer screenings for early prevention, especially since CCAC presentation is largely vaginal bleeding but still variable. 

The differential diagnoses for cervical neoplasms are broad, including microglandular hyperplasia, mesonephric adenocarcinoma, metastatic clear cell renal cell carcinoma, squamous cell carcinoma, and urothelial carcinoma, which require biopsy for diagnosis [5]. Appearance on histology is akin to clear cell carcinoma in other areas of the genital tract, including the endometrium, making an accurate diagnosis difficult [9]. This likely played a role in the initial misdiagnosis of clear cell endometrial adenocarcinoma that was used to guide initial intervention in our patient. Treatment of CCAC mirrors other types of cervical cancer, the standard of care being radical hysterectomy and pelvic lymphadenectomy in early stages [3,9]. After surgery, the pathological report can confirm the diagnosis. Immunohistochemistry markers Napsin A+/IMP3+ are sensitive for this clear cell carcinoma subtype [10,11,12], which were positive in our patient.

The etiology and disease mechanisms of CCAC are still largely unknown [13]. Risk factors for the most common cervical cancer subtypes, like squamous cell carcinoma, including HPV infection, smoking, and increased number of sexual partners, were not reportedly associated with CCAC [3,13]. Due to the uncertainty surrounding this cancer, current treatments are based on previous case reports, tumor size, and expert opinion [14], highlighting the need for further research into its clinicopathological features to ensure a proper approach to care.

## Conclusions

While clear cell adenocarcinoma of the cervix remains a rare entity, its association with diethylstilbestrol exposure underscores the importance of vigilant cervical cancer screening, particularly for DES daughters. However, as demonstrated in this case, CCAC can also occur in DES-independent patients, emphasizing the need to include it in the differential diagnosis for abnormal vaginal bleeding in this population. The rarity of this tumor, especially in patients without exposure to DES, along with its histological similarities with other malignancies, makes characterization challenging. Investigation into current case reports can help to elucidate uncertainty and guide therapeutic intervention. Encouraging timely cervical cancer screenings remains crucial for early detection and intervention, particularly given the variable presentation and potentially aggressive nature of this rare tumor.

## References

[1] Stelzle D, Tanaka LF, Lee KK (2021). Estimates of the global burden of cervical cancer associated with HIV. Lancet Glob Health.

[2] (2024). Diethylstilbestrol (DES) exposure and cancer. https://www.cancer.gov/about-cancer/causes-prevention/risk/hormones/des-fact-sheet.

[3] Mathew Thomas V, Alexander SA, Hadfield MJ, Vredenburgh J (2020). A rare case of clear cell adenocarcinoma of the cervix with no intrauterine diethylstilbestrol exposure. Cureus.

[4] Galic V, Herzog TJ, Lewin SN (2012). Prognostic significance of adenocarcinoma histology in women with cervical cancer. Gynecol Oncol.

[5] (2024). Clear Cell Carcinoma. https://www.pathologyoutlines.com/topic/cervixclearcell.html.

[6] Zoodsma M, Sijmons RH, de Vries EG, Zee AG (2004). Familial cervical cancer: case reports, review and clinical implications. Hered Cancer Clin Pract.

[7] Herbst AL (2000). Behavior of estrogen-associated female genital tract cancer and its relation to neoplasia following intrauterine exposure to diethylstilbestrol (DES). Gynecol Oncol.

[8] Reich O, Tamussino K, Lahousen M, Pickel H, Haas J, Winter R (2000). Clear cell carcinoma of the uterine cervix: pathology and prognosis in surgically treated stage IB-IIB disease in women not exposed in utero to diethylstilbestrol. Gynecol Oncol.

[9] Limaiem F, Cue L, Martingano D, Mandy H (2024). Clear Cell Carcinoma of the Cervix. StatPearls [Internet].

[10] Weidemann S, Böhle JL, Contreras H (2021). Napsin A expression in human tumors and normal tissues. Pathol Oncol Res.

[11] Lu L, Wang S, Zhu Q (2018). The expression of IMP3 in 366 cases with ovarian carcinoma of high grade serous, endometrioid and clear cell subtypes. Pathol Res Pract.

[12] Li C, Rock KL, Woda BA, Jiang Z, Fraire AE, Dresser K (2007). IMP3 is a novel biomarker for adenocarcinoma in situ of the uterine cervix: an immunohistochemical study in comparison with p16(INK4a) expression. Mod Pathol.

[13] Liu Y, Shi X, Yang J, Zhou H, Peng P, Cao D (2023). Clinical features and prognostic factors of cervical clear cell adenocarcinoma: a retrospective analysis of 74 cases from a tertiary hospital. Technol Cancer Res Treat.

[14] Haddout S, Imami Y, Benhessou M, Ennachit M, El Karroumi M (2022). Primary clear cell adenocarcinoma of the vagina not associated with diethylstilbestrol: a case report. Int J Surg Case Rep.

